# Postnatal health and care following hypertensive disorders in pregnancy: a prospective cohort study (BPiPP study)

**DOI:** 10.1186/s12884-022-04540-2

**Published:** 2022-04-05

**Authors:** Danielle C. Ashworth, Liza Bowen, Sophie P. Maule, Paul T. Seed, Marcus Green, Debra Bick, BPiPP study group, Lucy C. Chappell

**Affiliations:** 1 Department of Women & Children’s Health, King’s College London, London, UK; 2 Department of Population Health Sciences, King’s College London, London, UK; 3 Action on Pre-eclampsia, Charity, Evesham, Worcestershire UK; 4 Warwick Clinical Trials Unit, University of Warwick, Warwick, UK

**Keywords:** Pregnancy, Hypertension, High blood pressure, Postnatal

## Abstract

**Introduction:**

One in 10 women have hypertensive disorders in pregnancy (HDP) and are at risk of adverse short- and long-term health outcomes, yet there is limited information on their postnatal health and care needs. This study aimed to look at postnatal physical and psychological morbidity in women with HDP, compared to women without HDP, and the postnatal care received in both groups.

**Methods:**

Within a prospective cohort study, women with and without HDP were identified and recruited on the postnatal ward of 17 maternity units across England and invited to complete a short baseline questionnaire. At 3 months postpartum, women were sent a follow-up questionnaire, with reminders. The principal outcomes were the mean score at 3 months for the Edinburgh Postnatal Depression Scale (EPDS) and the EuroQol Group 5-dimension (EQ-5D) scale.

**Results:**

One thousand eight hundred twenty-nine women agreed to participate. Of these, 1757 (96%) completed the baseline questionnaire: 769 (44%) women had HDP and 988 (56%) women did not. Despite a difference in health-related quality of life and symptoms of depression at baseline between the two groups, at 3 months postnatal, within the 653 women who completed their follow-up questionnaire (37.2% of those who completed the baseline questionnaire) there were no significant differences between the groups (median EQ-5D VAS: 85 in women with HDP, 85 in women without HDP, *p* = 0.99 and mean EPDS score 5.5 in women with HDP, 5.0 in women without HDP, *p* = 0.80). Overall levels of physical postnatal morbidity were high, with 89% reporting one or more morbidities. Approximately 9% of women were re-admitted within 3 months after birth, higher in the HDP group (13.1%) higher compared to women without HDP (5.5%; RR 2.41; 95% CI 1.44–4.05).

**Conclusion:**

Overall levels of physical and psychological morbidity were high in this postnatal population. Although there were increased needs of women with HDP in the immediate postnatal period (compared to other women), their health assessments were similar at 3 months. This study highlights the unmet needs of women in the postnatal period, in addition to a missed opportunity to influence future pregnancies and improve the longer-term health of women and their babies.

**Supplementary Information:**

The online version contains supplementary material available at 10.1186/s12884-022-04540-2.

## Introduction

Hypertensive Disorders of Pregnancy (HDP) affect approximately 10% of all pregnancies [1] and include chronic hypertension (pre-existing hypertension present before 20 weeks’ gestation), gestational hypertension (hypertension presenting after 20 weeks’ gestation) and pre-eclampsia (de novo, or superimposed on chronic hypertension, with proteinuria and/or renal, hepatic or haematological dysfunction). HDP are associated with substantial maternal and perinatal morbidity and mortality [2–4], responsible for approximately 14% of maternal deaths globally [5]. There is also an increased risk of recurrence of HDP in future pregnancies [6] and increased lifetime risk of maternal cardiovascular disease [7, 8].

In the United Kingdom (UK), postnatal care aims to improve and support birth recovery and good maternal and infant physical and psychological health [9]. Most women commence postnatal care in hospital, and this continues at home or in community-based clinics with midwives and health visitors after hospital discharge. Women typically are offered a routine consultation with their family doctor (GP) at 6–8 weeks after birth. Although for many years this was primarily focused on a check of the infant, this has recently been mandated to also include a maternal health check [10]. Clinical guidance is provided for routine postnatal care for women and their infants [11] with guidance for women with HDP covered by the Hypertension in Pregnancy: diagnosis and management guideline [12].

Research to date has focused predominantly on antenatal management of HDP. The neglect of the postnatal period in the evidence base and lack of evidence of postnatal care being tailored to meet individual maternal need has been highlighted as a missed opportunity to improve wellbeing and reduce long-term adverse health outcomes for women and children [9, 13]. Although a number of large observational studies in the 1990s [14–16] highlighted extensive maternal postnatal morbidity, there has been a lack of focus in more recent years.

Given the sparsity of recent evidence on postnatal health and wellbeing, and extent to which current postnatal services reflect implementation of current guidance, this study aimed to address the postnatal morbidity at 3 months after birth for women with and without HDP, specifically including both physical and psychological conditions. We aimed to evaluate whether women with HDP experience greater postnatal physical and psychological morbidity than those without HDP, and the extent and characterisation of postnatal morbidity.

## Methods

### Study design and setting

In this prospective multicentre cohort study, women were recruited on the postnatal wards of 17 maternity units across England between February 2019 and March 2020. Women who were 18 years old or above and who gave birth at ≥20^+ 0^ weeks’ gestation were eligible. The study was registered with the ISRCTN registry (ISRCTN10809239) and approved by the London - South East Research Ethics Committee (REC reference: 18/LO/2084).

### Patient and public involvement

The Participant Information Sheet and study questionnaire were reviewed by members of the ‘Hypertension in Pregnancy Patient and Public Involvement group’ established at the lead site. Feedback forms demonstrated women’s support of the study (all completed forms indicated women felt the study to be ‘very important’ and that they would likely take part if invited) and no concerns were raised about the usefulness, length or clarity of the questionnaire or Participant Information Sheet. Issues were discussed relating to potential barriers to recruitment. Feedback from these meetings led to adjustments in study design, Participant Information Sheet content, questionnaire content, and questionnaire delivery method (online was unanimously preferred over paper and postal routes).

### Recruitment and data collection

Eligible women were identified using the postnatal ward list at participating maternity units. A convenience sample of these women were approached by local research midwives, based on aiming to recruit similar numbers of women who were hypertensive and normotensive. Eligible women who wished to take part in the study provided their written electronic consent.

Recruited participants were asked to complete an initial (baseline) questionnaire on the postnatal ward and a follow-up questionnaire at 12 weeks postpartum, emailed (or posted to women if they preferred). The questionnaires captured information on their obstetric and medical history, family history of obstetric complications and relevant medical conditions, demographic information, infant feeding, current physical and mental health, postnatal care received, and their plans for future pregnancies. Specific morbidities included: extreme tiredness/ exhaustion; severe headaches/ migraines; back pain; perineal wound infection; Caesarean wound infection; breastfeeding problems; leakage of urine; leakage of stool; or other problems (as provided in free text answers). Both questionnaires included validated health measures: Edinburgh Postnatal Depression Scale (EPDS) (10 questions used to detect possible postnatal depression) [17], EuroQol Group five dimension (EQ-5D) (two-page assessment of health-related quality of life, consisting of five dimensions each with levels of severity and a vertical visual analogue scale) [18, 19] and the Whooley questions (two questions used to routinely screen for maternal mental health issues during and after pregnancy in NHS maternity services) [20]. Women whose EPDS score was 13 or over (i.e., indicating a presence of possible depressive symptoms) or women who answered positively to question 10 (i.e., indicating potential self-harm or suicidal thoughts) were either flagged to their case midwife on the postnatal ward (following baseline questionnaire completion) or contacted by the central research team (on follow-up questionnaire completion). At follow-up, the central research team ensured these women were aware of the services provided by appropriate healthcare professionals (e.g., health visitor, midwife, family doctor). Following discussion with Patient and Public Involvement representatives, all women were offered the opportunity to complete follow-up, even if they had not had a live birth.

Research midwives entered additional participant data directly into the study’s secure online central database (MedSciNet, Stockholm, Sweden) extracted from the maternity records (with the participant’s consent).

### Statistical analysis

Data analysis was conducted in Stata version 16 [21]. Means and frequency tables were reported and differences between the groups (women with HDP and women without HDP) examined using t-test, chi-square statistics, and median differences analysed through median tests. Differences between the groups for binary outcomes were examined using binary regression with a log link to give Risk Ratios. For the principal outcomes, we adjusted for drop-out at the 3-month timepoint by using inverse probability weightings to correct for potential imbalance in the case mix seen at enrolment. Models were not adjusted for confounders as we were interested in outcome prevalence rather than mechanisms. Adjusting for variables at enrolment could have removed consequences of pregnancy hypertension we were seeking to describe. In line with recent literature, an EPDS cut-off of 13 or more was used to identify women for further assessment for depression [22].

### Free text analysis

To supplement the quantitative results and in response to initial Patient and Public Involvement work, the follow-up questionnaire included three open-ended questions. Participants were asked whether they had any comments about their health and wellbeing since having their baby; whether they had any comments about their postnatal care; and whether their perinatal experiences had changed their opinion on future pregnancies. Data were managed and analysed in NVivo 12 [23], using a thematic framework analysis. The most illustrative quotations are presented in the results section.

## Results

**Fig. 1:**
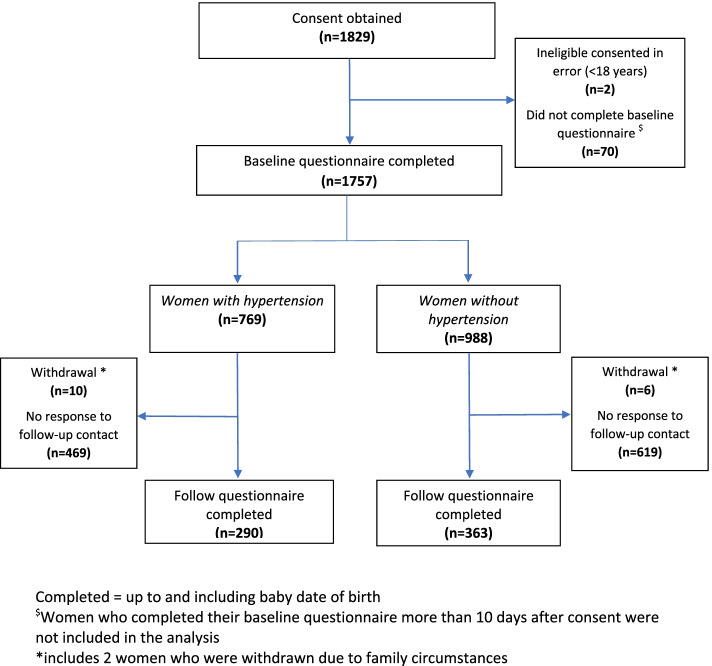
Flow of participants

Of the 2893 eligible women approached, 1829 (63%) consented to take part. Of these, 1757 (96%) completed the baseline questionnaire (at postnatal enrolment): 769 (44%) women had HDP and 988 (56%) women did not. Follow-up questionnaires (at 12 weeks postnatal) were completed by 653 of the 1757 (37%) women: 290 of 769 women (38%) with HDP and 363 of 988 women (37%) without HDP (Fig. 1).

Characteristics of the study population at postnatal enrolment are shown in Tables 1 and 2, supplementary Table 1 and supplementary Table 2; differences are noted between the two groups (women with HDP and women without HDP) in ethnicity, risk factors for hypertension, Body Mass Index, parity, multi-fetal births, intended and actual mode of birth, labour analgesia, maternal and neonatal length of inpatient postnatal stay, admission to Neonatal Unit, and proportion of small-for-gestational age infants.

**Table 1:** Self-reported maternal demographics at postnatal enrolment

	Total	Hypertensive in pregnancy	Normotensive in pregnancy	Comparison/Difference
**Number of women (*n*, %)**	**1757**	**769 (43.8)**	**988 (56.2)**	
**Maternal age, years (mean, SD)**	32.7 (5.5)	32.9 (5.8)	32.6 (5.2)	0.3538
**Ethnicity groupings**				
Black/Black mixed	263 (15.0)	145 (18.9)	118 (11.9)	< 0.0001
Asian/Asian mixed	187 (10.7)	78 (10.1)	109 (11.0)	
White	1230 (70.0)	508 (66.1)	722 (73.1)	
Other	75 (4.3)	37 (4.8)	38 (3.9)	
Missing	2 (0.1)	1 (0.1)	1 (0.1)	
**First baby**	1076 (61.3)	501 (65.2)	575 (58.2)	0.003
**Singleton baby**	1712 (97.4)	739 (96.1)	973 (98.5)	0.002
**Self-reported hypertension type***(non-exclusive)*				
Chronic		84 (10.9)	Not applicable	
Gestational		240 (31.2)		
Pre-eclampsia		311 (40.4)		
Eclampsia		6 (0.8)		
HELLP syndrome		15 (2.0)		
Hypertension (not specified)		202 (26.3)		
**Education level beyond secondary**	1375 (78.3)	587 (76.3)	788 (79.8)	0.086
**Currently employed (self-reported)**	1321 (75.2)	569 (74.0)	752 (76.1)	0.311
**Living with others**	1554 (88.5)	668 (86.9)	886 (89.7)	0.069
**Any risk factors for hypertension**	1395 (79.4)	684 (89.0)	711 (72.0)	< 0.0001
**Any mental health condition**	307 (17.5)	149 (19.4)	158 (16.0)	0.064
**Intended mode of birth**				
Spontaneous vaginal birth	1062 (60.4)	421 (54.8)	641 (64.9)	
Elective c-section	304 (17.3)	117 (15.2)	187 (18.9)	
Planned induction (vaginal birth)	289 (16.5)	157 (20.4)	132 (13.4)	
Not discussed - delivered early	24 (1.4)	20 (2.6)	4 (0.4)	
Not discussed – other	78 (4.4)	54 (7.0)	24 (2.4)	
**At initial antenatal visit**				
Body mass index, kg/m^2^ (median, IQR)	26 (22.5–30.7)	27 (23.4–32.5)	25 (22–29)	< 0.0001
Current smoker	97 (5.5)	46 (6.0)	51 (5.2)	0.458
**Positive to either Whooley question**	124 (7.1)	64 (8.3)	60 (6.1)	OR 1.39 (0.97–2.02)
Missing	134 (7.6)	56 (7.3)	78 (7.9)	

*HELLP* Haemolysis, Elevated Liver enzymes, Low Platelets, *OR* odds ratio

**Table 2:** Characteristics of labour, birth, and post-delivery postnatal stay at postnatal enrolment

	Totals	Hypertensive in pregnancy	Normotensive in pregnancy	Comparison/Difference
**Maternal outcomes: number of women (n, %)**	**1757**	**769 (43.8)**	**988 (56.2)**	
Use of regional anaesthetic in labour	1228 (69.9)	572 (74.4)	656 (66.4)	< 0.0001
Second/third/fourth degree perineal tear or episiotomy	668 (38.0)	250 (32.5)	418 (42.3)	0.060
Length of postnatal inpatient stay, days (median, IQR)	3 (2–4)	3 (2–5)	2 (1–3)	< 0.0001
Admission to either high dependency unit or intensive care unit^b^	181(10.3)	86 (11.2)	95 (9.6)	0.287
Length of stay in high dependency unit or intensive care unit, days (median, IQR)	1 (1–1)	1 (1–2)	1 (1–1)	< 0.0001
**Perinatal outcomes: number of infants**	**1803**	**799 (44.3)**	**1004 (55.7)**	
Live births	1802 (99.9)	799 (100.0)	1003 (99.9)	0.372
**Mode of birth**				
Vaginal	657 (36.4)	252 (31.5)	405 (40.3)	< 0.0001
Forceps	168 (9.3)	68 (8.5)	100 (10.0)	
Ventouse	137 (7.6)	55 (6.9)	82 (8.2)	
Pre-labour Caesarean section	462 (25.6)	236 (29.5)	226 (22.5)	
In-labour Caesarean section	379 (21.0)	188 (23.5)	191 (19.0)	
**Gestational age at birth, weeks (mean, SD)**	38.8 (2.3)	38.2 (2.6)	39.4 (1.9)	< 0.0001^a^
**Birthweight, kg (mean, SD)**	3.198 (0.671)	3.010 (0.743)	3.348 (0.565)	< 0.0001^a^
**Small for Gestational Age (<10th birthweight centile)**	184 (10.2)	177 (14.6)	67 (6.7)	< 0.0001
**Length of infant inpatient stay, days (mean, SD)**	4.2 (6.7)	5.7 (9.1)	3.1 (3.5)	< 0.0001^a^
**Baby (ies) admitted to the neonatal unit**	230 (12.8)	155 (19.4)	75 (7.5)	< 0.0001
**Length of stay in neonatal unit, days (mean, SD)**	9.7 (9.6)	11.5 (9.9)	5.9 (7.6)	< 0.0001^a^

*OR* odds ratio

^a^Adjusted for clustering by twins

^b^Includes post Caesarean section recovery

### Postnatal enrolment

**Fig. 2:**
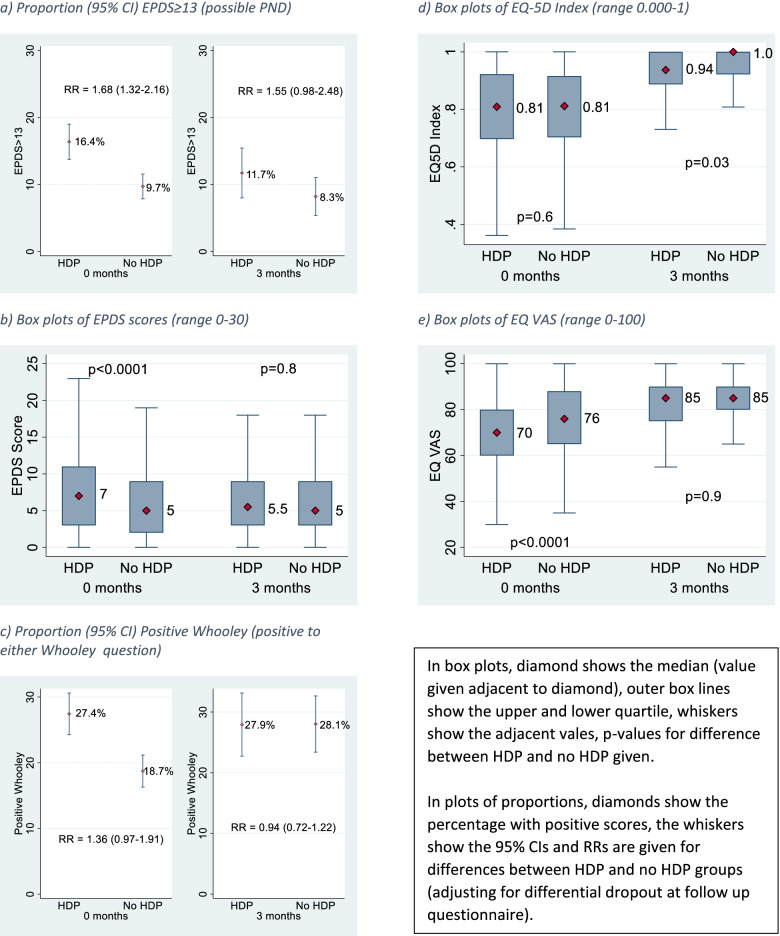
Mental and physical health scores in women with and without hypertensive disorders of pregnancy, at 0 months (*n* = 769 with HDP; *n* = 988 with no HDP) and 3 months (*n* = 290 with HDP; *n* = 363 with no HDP) postnatally. **a** Proportion (95% CI) EPDS≥13 (possible PND). **b** Box plots of EPDS scores (range 0–30). **c** Proportion (95% CI) Positive Whooley (positive to either Whooley question). **d** Box plots of EQ-5D Index (range 0.000–1). **e** Box plots of EQ VAS (range 0–100)

At postnatal enrolment, health-related quality of life, as measured by the EQ-5D Visual Analogue Scale, was lower in women with HDP compared with women without HDP (median EQ-5D VAS: 70 in women with HDP, 76 in women without HDP) but not in the EQ-5D index score between the groups (Fig. 2, supplementary Table 3). Symptoms of depression were also higher in the HDP group; for the EPDS, there was a median score of 7 (with 16.4% having scores ≥13) in women with HDP compared to a median score of 5 (with 9.7% having scores ≥13) in women without HDP (Risk Ratio 1.68; 95% CI 1.32–2.16). For the Whooley score, 27.4% of the women with HDP were positive to either question one or two compared to 18.7% of women without HDP, (RR 1.36; 95% CI 0.97–1.91) (Fig. 2, supplementary Table 3).

### Three month postnatal physical and mental wellbeing

At 3 months postnatal, there was no evidence of a significant difference between the groups in physical and mental health scores, including the mean Edinburgh Postnatal Depression Scale score (*p* = 0.80), median EQ-5D quality of life scale score (*p* = 0.99), the Whooley responses (RR 0.94; 95% CI 0.72–1.22) and most physical morbidities (Fig. 2; Table 3).

**Table 3:** Main primary and secondary outcomes at 3 months postnatal

	Totals	Hypertensive in pregnancy	Normotensive in pregnancy	Comparison/difference
**Number of women**	**653**	**290 (44.4)**	**363 (55.6)**	
EPDS score ≥ 13	64 (9.8)	34 (11.7)	30 (8.3)	OR 1.47 (0.88–2.47)
EPDS total score (mean, SD)	6.2 (4.6)	6.3 (4.8)	6.1 (4.5)	0.617
Positive to either Whooley Q1 or Q2	183 (28.0)	81 (27.9)	102 (28.1)	OR 0.99 (0.70–1.40)
EQ-5D index score (mean, SD)	0.935 (0.088)	0.926 (0.093)	0.943 (0.084)	0.0222
EQ-5D VAS score (0–100)	82.8 (13.3)	82.2 (13.5)	83.2 (13.1)	0.3557
Any reported morbidity at 3 months postnatal	582 (89.1)	260 (89.7)	322 (88.7)	OR 1.17 (0.49–2.77)
Women with any morbidity who consulted a health care professional	378 (57.9)	171 (59.0)	207 (57.0)	OR 1.19 (0.50–2.86)
Morbidities reported:				
Extreme tiredness/ exhaustion	252(38.6)	120 (41.4)	132 (36.4)	OR 1.23 (0.90–1.69)
Severe headaches/ migraines	100 (15.3)	55 (19.0)	45 (12.4)	OR 1.65 (1.07–2.53)
Back pain	357 (54.7)	168 (57.9)	189 (52.1)	OR 1.26 (0.92–1.72)
Perineal wound infection	36 (5.5)	18 (6.2)	18 (5.0)	OR 1.33 (0.67–2.63)
Caesarean wound infection	61 (9.3)	33 (11.4)	28 (7.7)	OR 1.22 (0.70–2.14)
Breastfeeding problems	298 (45.6)	120 (41.4)	178 (49.0)	OR 0.69 (0.50–0.96)
Leakage of urine	189 (29.0)	83 (28.6)	106 (29.2)	OR 0.97 (0.69–1.36)
Leakage of stool	26 (4.0)	10 (3.5)	16 (4.4)	OR 0.77 (0.35–1.73)
Other problems	161 (24.7)	73 (25.2)	88 (24.2)	OR 1.05 (0.73–1.50)

*EPDS* Edinburgh Postnatal Depression Scale, *EQ-5D VAS* EuroQol Group five dimension visual analogue scale, *OR* odds ratio

Overall levels of physical postnatal morbidity were high, with 89% reporting one or more morbidities since birth, with back pain (55%) and breastfeeding problems (46%) most commonly reported (Table 3). Rates of responses to ‘ever breastfed’ or ‘currently breastfeeding’ at 3 months were similar in both groups. For breastfeeding, 90% reported having ever breastfed and 52% were currently breastfeeding (Table 4, supplementary Table 4).

**Table 4:** Postnatal care at 3 months postnatal and opinions on future pregnancies (self-reported)

	Totals	Hypertensive in pregnancy	Normotensive in pregnancy	Comparison/ difference
**Number of women**	**653**	**290 (44.4)**	**363 (55.6)**	
**Breastfeeding**				
Ever breastfeed baby	586 (89.7)	261 (90.0)	325 (89.5)	OR 1.05 (0.63–1.75)
First feed breastmilk (of those who ever breastfed)	540 (92.2)	231 (88.5)	309 (95.2)	OR 0.40 (0.21–0.75)
Currently feeding breastmilk (of those who ever breastfed)	305 (52.1)	129 (49.4)	176 (54.2)	OR 0.83 (0.60–1.15)
**BP measured in hospital**	619 (94.8)	274 (94.5)	345 (95.0)	
**Frequency of BP measured in hospital (where measured)**				
Less than once a day	33 (5.3)	7 (2.6)	26 (7.5)	< 0.0001
Approximately once a day	115 (18.6)	23 (8.4)	92 (26.7)	
More than once a day	460 (74.3)	240 (87.6)	220 (63.8)	
Other	11 (1.8)	4 (1.5)	7 (2.0)	
**BP medication taken between birth and 6 weeks postnatal**	208 (31.9)	194 (66.9)	14 (3.9)	OR 54.17 (20.0–97.80)
**One or more postnatal visits at:**				
Home	505 (77.3)	236 (81.4)	269 (74.1)	OR 1.70 (1.14–2.52)
Hospital/ clinic	215 (32.9)	104 (35.9)	111 (30.6)	OR 1.30 (0.94–1.81)
GP	466 (71.4)	222 (76.6)	244 (67.2)	OR 1.73 (1.20–2.48)
**Median (IQR) number of postnatal visits (of women visited) at:**				
Home	2 (2–3)	3 (2–2)	3 (4–2)	0.174
Hospital/ clinic	2 (3–1)	2 (3–1)	1 (2–1)	0.553
GP	1 (2–1)	2 (3–1)	1 (2–1)	< 0.0001
**BP never measured at postnatal visits (of women visited) at:**				
Home	196 (38.8)	56 (23.7)	140 (52.0)	< 0.0001
Hospital/ clinic	77 (35.8)	24 (23.1)	53 (47.8)	< 0.0001
GP	90 (19.3)	23 (10.4)	67 (27.5)	< 0.0001
**Readmitted to hospital**	58 (8.9)	38 (13.1)	20 (5.5)	OR 2.6 (1.49–4.64)
**Readmitted to hospital due to BP**	28 (4.3)	24 (8.3)	4 (1.1)	OR 8.10 (2.78–23.62)
**Attendance at GP postnatal check**	584 (89.4)	258 (89.0)	326 (89.8)	OR 1.06 (0.61–1.84)
**BP measured at GP postnatal check (of those attending)**	459 (78.6)	214 (83.0)	245 (75.2)	OR 1.61 (1.07–2.42)
**Any self-monitoring of BP since birth**	154 (23.6)	125 (43.1)	29 (8.0)	OR 9.03 (5.78–14.12)
**Regular self-monitoring of BP since birth**	79 (12.1)	72 (24.8)	7 (1.9)	OR 16.80 (7.59–37.16)
**Excellent/Very Good/Good opinion of postnatal care**	548 (83.9)	232 (80.0)	316 (87.1)	OR 0.63 (0.40–0.98)
**Women likely to consider having a future pregnancy**	421 (64.5)	192 (66.2)	229 (63.1)	OR 1.19 (0.86–1.66)
**Women currently pregnant**	3 (0.5)	2 (0.7)	1 (0.3)	OR 2.54 (0.23–28.19)
**Women who have changed their family plans**	206 (31.6)	107 (36.9)	99 (27.3)	OR 1.59 (1.14–2.23)

*BP* blood pressure, *GP* general practitioner, *OR* odds ratio

Although temporal trends are visible in Fig. 2, the number of women completing the follow-up questionnaire at 3 months postnatal was substantially smaller (653 women) than the group completing the baseline questionnaire (1751 women).

Analysis of free-text responses (106 from women with HDP, 116 from women with no HDP; Table 5) regarding health and wellbeing in the postnatal period identified both physical and mental health as common themes in both groups: *“I was a little disappointed to have to seek help myself in issues with my mental health. I do believe it was due to how I was spoken to and treated by the healthcare professionals…. Now I have sought help myself, I feel much better and enjoying motherhood”* (woman with HDP). Another theme that developed was around the support available: *“After the regular appointments in pregnancy, you feel rather abandoned after birth”* (woman with no HDP).

**Table 5:** Thematic framework analysis of free-text open-ended questions

Themes	Total	Hypertensive in pregnancy	Normotensive in pregnancy
**Aspects of recent maternity care that have influenced decision-making around future pregnancies**			
**Number of women responding**	206	107 (51.9)	99 (48.1)
1. Caesarean section	21 (10.2)	16 (15.0)	5 (5.1)
2. Content with care received	24 (11.7)	15 (14.0)	9 (9.1)
3. Induction of labour	3 (1.5)	0 (0.0)	3 (3.0)
4. Hypertensive disorders of pregnancy	25 (12.1)	25 (23.4)	0 (0.0)
5. Lessons for the future (advocating, change in care, preparedness)	37 (18.0)	21 (19.6)	16 (16.2)
6. Put off by experience/postnatal care	29 (14.1)	13 (12.1)	16 (16.2)
**Highlighted aspects of postnatal health and well-being**			
**Number of women responding**	222	106 (47.7)	116 (52.3)
1. Blood pressure and postnatal care	36 (16.2)	36 (34.0)	0 (0.0)
2. Breastfeeding	10 (4.5)	0 (0.0)	10 (8.6)
3. Mental health	26 (11.7)	13 (12.3)	13 (11.2)
4. Physical health	29 (13.1)	13 (12.3)	16 (13.8)
5. Support (including by health care professionals)	19 (8.6)	12 (11.3)	7 (6.0)
**Views on postnatal care**			
**Number of women responding**	272	129 (47.4)	143 (52.6)
1. Breastfeeding	29 (10.7)	11 (8.5)	18 (12.6)
2. GP/community care	72 (26.5)	39 (30.2)	33 (23.1)
3. Hospital care	9 (3.3)	0 (0.0)	9 (6.3)
4. Lack of information	20 (7.4)	16 (12.4)	4 (2.8)
5. Postnatal ward	66 (24.3)	37 (28.7)	29 (20.3)

*GP* general practitioner

### Self-reported postnatal care

Significant differences between the groups in self-reported postnatal care were seen in blood pressure evaluations and medication use in and out of hospital (Table 4, supplementary Table 5). Approximately 9% of women were readmitted to hospital within 3 months postnatally, with re-admission in women with HDP higher (RR 2.41; 95% CI 1.44–4.05) compared with women without, including for blood pressure concerns (RR 7.71; 95% CI 2.69–22.12). One in four women self-monitored their blood pressure postnatally; 43% of women with HDP had ever measured their BP postnatally (compared to 8% of women without HDP; RR 5.47; 95% CI 3.77–7.94) and 25% of women with HDP regularly measured their BP (compared to 2% of women without HDP).

Women’s opinion of their postnatal care was generally high with 84% of all women reporting ‘excellent’, ‘very good’, or ‘good’ care (Table 4, supplementary Table 5).

Analysis of free-text responses (129 from women with HDP, 143 from women without HDP, Table 5) regarding postnatal care identified postnatal ward care as a theme: *“The hospital care postnatal was good but felt that the ward was understaffed, especially in regard to support for infant feeding”* (*w*oman with no HDP). Primary care and community care were another theme: some women reported positive experiences with family doctors, but several felt that the 6-week postnatal check felt rushed or brief: *“I think the check I had at my GP* [primary care/family physician] *after 6 weeks was not really thorough and when I tried to book an appointment afterwards because I have had pain I couldn’t get one until 3 weeks after and then it was cancelled the day before without giving me a new appointment”* (woman with HDP). A third theme was breastfeeding, with references to support both in hospital and in the community: *“I feel if the NHS wants to push breastfeeding as the best option for babies and mothers, they need to provide much more aftercare support”* (woman with no HDP). The final theme was a lack of or conflicting information, particularly in the HDP group: *“I wish I had more information on the impact that high blood pressure can have on my future health and potential pregnancies”* (woman with HDP).

On being asked about plans for future pregnancies, 37% of women with HDP had changed their family plans following birth, compared with 27% in women without HDP) (Table 4, supplementary Table 5). This was reflected in free-text responses (107 from women with HDP, 99 from women with no HDP, Table 5): *“I’m now scared to become pregnant again, I always wanted more than one child now I’m worried and l will develop pre-eclampsia again”* (woman with HDP). In both groups, women also talked more generally about how aspects of their experiences made them less likely to consider becoming pregnant again, but there were also positive comments about lessons for the future. “*I would be more aware of postnatal problems. But more aware now that even a not so positive birth is still amazing when you have your baby”* (woman with no HDP).

An analysis of maternal demographics, characteristics of birth, and study-specific outcomes captured at postnatal enrolment by responder status (with women defined as ‘responders’ if they had completed the follow-up questionnaire at 3 months postnatal) is shown in supplementary Tables 6, 7 and 8. Whilst the groups are similar in many characteristics, responding participants were more likely (than non-responders) to be of White ethnicity, educated to beyond secondary school level, and to have given birth to their first baby.

## Discussion

In this prospective cohort study of women with and without HDP, we found overall postnatal physical and psychological morbidity at 3 months postpartum to be high, with around 90% of the entire cohort reporting some postnatal morbidity at 3 months after birth. There were significant differences in postnatal physical and mental health scores between women with and without HDP in the immediate postnatal period, but these differences converged by 3 months. Screening for symptoms of postnatal depression, using the Whooley questions, showed that at initial antenatal visit, one in fourteen women screened positively (this was higher in women with HDP compared with women without HDP); however, at immediately postnatal nearly one in four women screened positively (significantly higher in women with HDP compared with women without HDP) and then nearly a third of women at 12 weeks postnatal (with no significant differences between the groups). Approximately one in 10 women in this study were readmitted to hospital within the first 3 months after birth, with post birth infections the most commonly reported reason. Approximately 1 in 20 were readmitted due to their blood pressure. Women with HDP were more likely to be readmitted compared to women without, particularly with blood pressure as the reason for admission. In around a quarter of postnatal visits in women with HDP, and around half of visits in women without HDP, women did not have their blood pressure measured. Women with HDP were more likely to change their future pregnancy plans. Some statistically significant differences in outcomes are likely to be under the threshold of a minimally important clinical difference for this population group, such as differences in median EQ-5D visual analogue scale scores. Our sensitivity analysis found that characteristics associated with responder status were in line with other literature. Any significant differences in characteristics by response status were not substantial enough to have invalidated our study findings.

Strengths of this study include the large sample size of diverse women, across a number of maternity units across England (representative of the wider pregnancy population), contributing to the generalisability of the findings in similar health-care settings. The study included women with and without HDP as well as addressing women’s physical and mental co-morbidities together. Further, the study used electronic consent and primarily electronic data capture, a mode of capture appropriate for this population. Ninety-nine percent of women between 16 and 44 years old report ‘daily’ or ‘almost daily’ use of the internet, and 98% report using their mobile phone and smartphone as the most popular device to access the internet on the go [24].

Limitations of the study include the convenience sampling of eligible women identified on the postnatal ward at participating maternity units and therefore there may have been some selection bias in the study population. The study had lower than anticipated retention rates at 3 months postnatal (37% response rate). Although the study team employed strategies to reduce loss to follow-up and used a diverse range of contact options for questionnaire completion reminders, most women did not complete the 3-month follow-up questionnaire. Although smaller studies undertaken by a single dedicated researcher may have higher completion rates, this study was intentionally designed as a large multicentre cohort study. The completion rate is likely to reflect the many competing demands during a woman’s postnatal period and the impact of this on research in the postnatal period, using this methodology, needs to be considered.

A recent review of postnatal mental health following HDP [25] concluded that women with HDP may be at increased risk of developing postpartum depression, anxiety, and post-traumatic stress disorder. Included within this review, Chen et al [26] reported a substantially higher prevalence of postnatal depression than our study, at 27% of 90 women with pre-eclampsia. This discrepancy may be explained by the differences in timing of postnatal data capture (6 weeks compared with 12 weeks) and the EPDS cut-off used (10 or higher compared with 13 or higher). Mautner et al [27] reported similar mean EPDS scores in the immediate postnatal period (EPDS mean 7.83 for women with HDP); however, at 3–4 months postpartum these scores were much lower (EPDS mean 3.67) than we report, a difference that may be explained by the small sample size of 18 women with hypertensive disorders in the study. Notably in our study we had a much larger sample size across a more diverse population, such that we are better able to interpret our findings.

The prevalence of HDP is expected to rise with increasing numbers of pregnant women presenting with risk factors, including older maternal age and obesity [12]. Despite changes in maternal characteristics or case-mix with more complex births, the length of inpatient postnatal stay in the UK is shortening. A study comparing postnatal stay in 92 countries (10 low-income, 42 middle-income and 40 high-income countries) reported that the UK had the shortest mean length of stay for singleton, vaginal births amongst high-income countries [28]. In addition to decline in duration of inpatient stay in UK settings, there has been a reduction in midwifery community-based contacts, likely to reflect reduced healthcare resources rather than maternal need or preference. The implications and impact of reduced postnatal care for both the woman and infant are currently unknown. The most recent UK Confidential Enquiry into Maternal Deaths and Morbidity [29] highlighted that pregnant and postnatal women are still dying from the complications of severe hypertension and the report recommendations reiterate the importance of controlling blood pressure in pregnancy and postpartum.

A 2009–2010 postnatal care survey of first-time mothers conducted by the National Childbirth Trust (*n* = 1260) documented differing standards of postnatal care and provision as a key issue. This report highlighted women’s lack of involvement in their postnatal care planning and understaffing of postnatal wards (42% of women said there were ‘sometimes or never’ enough midwives to provide them with the care and support on the hospital postnatal ward). In the first month after birth, women highlighted lack of emotional support (61% of women), insufficient physical care (50% of women), inadequate information and advice provided (59% of women) and insufficient support or conflicting advice on infant feeding (55% of women) [30]. This is further supported by one of the key findings of the 2019 Care Quality Commission maternity services survey (*n* = 17,151) which indicated a poorer experience of postnatal care compared with antenatal and intrapartum care, a finding which is in line with previous years [31]. Although not directly comparable with our study, these findings complement our results with the high level of postnatal morbidity reported by women. A 2013 Royal College of Midwives audit of midwives’ views (*n* = 2349) reported challenges around the provision of postnatal care, citing organisational pressures as the most influential factor in determining the number of postnatal contacts [32].

Our study highlights this ongoing unmet need in the postnatal period as identified by women, with a missed opportunity for intervention in this period to influence future pregnancies and cardiovascular health, the need to unite physical and mental health, and the challenges of undertaking studies involving postnatal women. Notably around 10% of women reported not attending their postnatal check at 6–8 weeks after birth, highlighting an important gap for these women. Although we found that electronic delivery of study information and questionnaires was acceptable and feasible, in line with other studies [33, 34], this does not overcome the difficulties of reaching women at this time.

Given the study findings, future research should further explore models of postnatal care (contacts and content) starting from initial antenatal visit, how women’s pregnancy health history and plans for care going forward are better communicated to primary care teams and ensuring that women are provided with information around risks, with the opportunity for questions to be addressed about their future health, including future pregnancies. These research recommendations complement a number of the top 10 prioritised research questions documented by the James Lind Alliance priority setting partnership on Hypertension in Pregnancy, including ‘How can we provide better support for women with pregnancy hypertension and their families?’, in which research questions were prioritised by women with lived experience of pregnancy hypertension and healthcare professionals [35]. Despite a recent major policy review of maternity care in England [13] and a specific recommendation in the NHS Long-Term Plan to improve perinatal pelvic floor health [36], much more work is needed to address the gaps in the provision of postnatal care to better meet maternal health needs.

In summary, this study usefully highlights the ongoing current gaps in clinical care and research needs for women with HDP in the postnatal period. If these gaps are to be addressed, review of postnatal policy and research funding priorities are urgently needed.

## Supplementary Information

**Additional file 1: Supplementary Table 1.** Maternal demographics at postnatal enrolment. **Supplementary Table 2.** Labour, birth, and postnatal characteristics at postnatal enrolment. **Supplementary Table 3.** Study specific postnatal enrolment baseline characteristics. **Supplementary Table 4.** Primary and main secondary outcomes at 3 months postnatal. **Supplementary Table 5.** Postnatal care at 3 months postnatal and opinions on future pregnancies (self-reported). **Supplementary Table 6.** Maternal demographics at postnatal enrolment by responder status. **Supplementary Table 7.** Characteristics of labour, birth and post-delivery postnatal stay at postnatal enrolment by responder status. **Supplementary Table 8.** Study specific outcomes at postnatal enrolment by responder status.

## Data Availability

The dataset generated during the current study will be available to appropriate academic parties on request from the Chief Investigator (LCC) in accordance with the data sharing policies of King’s College London.

## Abbreviations

HDP: Hypertensive Disorders of Pregnancy
EPDS: Edinburgh Postnatal Depression Scale
EQ-5D: EuroQol Group five-dimension scale
