# Supplementary material for: Postnatal health and care following hypertensive disorders in pregnancy: a prospective cohort study (BPiPP study)
Source: BMC Pregnancy Childbirth. 2022 Apr 5;22:286. doi: 10.1186/s12884-022-04540-2 (PMC8985263; doi:10.1186/s12884-022-04540-2)
Supplement: Supplementary file 1 — Additional file 1: Supplementary Table 1. Maternal demographics at postnatal enrolment. Supplementary Table 2. Labour, birth, and postnatal characteristics at postnatal enrolment. Supplementary Table 3. Study specific postnatal enrolment baseline characteristics. Supplementary Table 4. Primary and main secondary outcomes at 3 months postnatal. Supplementary Table 5. Postnatal care at 3 months postnatal and opinions on future pregnancies (self-reported). Supplementary Table 6. Maternal demographics at postnatal enrolment by responder status. Supplementary Table 7. Characteristics of labour, birth and post-delivery postnatal stay at postnatal enrolment by responder status. Supplementary Table 8. Study specific outcomes at postnatal enrolment by responder status. [file 12884_2022_4540_MOESM1_ESM.docx]

## Supplementary table 1: Maternal demographics at postnatal enrolment

|  | **Totals** | **Hypertensive**  **in pregnancy** | **Normotensive in pregnancy** |
| --- | --- | --- | --- |
| **Number of women** | **1757** | **769 (43.8)** | **988 (56.2)** |
| **Self-reported highest educational qualification**  None  O levels/GCSEs  AS/A levels/Highers/Advanced highers  Degree / Higher Degree  Other  Missing | 52 (3.0)  292 (16.6)  293 (16.7)  1082 (61.6)  36 (2.1)  2 (2.1) | 25 (3.3)  139 (18.1)  134 (17.4)  453 (58.9)  17 (2.1)  1 (0.1) | 27 (2.7)  153 (15.5)  159 (16.1)  629 (63.7)  19 (1.9)  1 (0.1) |
| **Self-reported employment status**  Employed full-time (including maternity leave)  Employed part-time (including maternity leave)  Self-employed  Student  Homemaker  Long-term disabled or sick  Unemployed  Retired  Other  Missing | 1065 (60.6)  256 (14.6)  56 (3.2)  30 (1.7)  184 (10.5)  14 (0.8)  140 (8.0)  0 (0.0)  10 (0.6)  2 (0.11) | 469 (61.0)  100 (13.0)  27 (3.5)  12 (1.6)  73 (9.5)  8 (1.0)  72 (9.4)  0 (0.0)  7 (0.9)  1 (0.1) | 596 (60.3)  156 (15.8)  29 (2.9)  18 (1.8)  111 (11.2)  6 (0.6)  68 (6.9)  0 (0.0)  3 (0.3)  1 (0.1) |
| **Self-reported partnership status**  Single  Married / Civil Partnership  Divorced / Separated  Partner deceased  Other  In a partnership  Other not categorised  Missing | 311 (17.7)  1247 (71.0)  13 (0.7)  2 (0.1)  182 (10.4)  178  4  2 (0.1) | 154 (20.0)  532 (69.2)  5 (0.7)  0 (0.0)  77 (10.0)  75  2  2 (0.1) | 157 (15.9)  715 (72.4)  8 (0.8)  2 (0.2)  105 (10.6)  103  2  1 (0.1) |
| **Self-reported risk factors for hypertension** *(non-exclusive)*  Any risk factor  First pregnancy  Gap 10 years  Multifetal pregnancy  Renal disease  Diabetes  Autoimmune disease  Other chronic condition  Personal previous high BP (outside of pregnancy)  Personal previous high BP (in previous pregnancy)  Family history of hypertension in pregnancy  Family history of hypertension | 1395 (79.4)  930 (52.9)  72 (4.1)  45 (2.6)  19 (1.1)  194 (11.0)  35 (2.0)  56 (3.2)  96 (5.5)  140 (8.0)  232 (13.2)  453 (25.8) | 684 (89.0)  447 (58.1)  40 (5.2)  29 (3.8)  13 (0.6)  95 (12.4)  18 (2.3)  25 (3.3)  85 (11.1)  112 (14.6)  141 (18.3)  248 (32.3) | 711 (72.0)  483 (48.9)  32 (3.2)  16 (1.6)  6 (1.7)  99 (10.0)  17 (1.7)  31 (3.1)  11 (1.11)  28 (2.8)  91 (9.2)  205 (20.8) |
| **Self-reported mental health condition** *(non-exclusive)*  Any condition  Anxiety and/or depression  Other mental health conditions | 307 (17.5)  296 (16.9)  43 (2.5) | 149 (19.4)  142 (18.5)  26 (3.4) | 158 (16.0)  154 (15.6)  17 (1.7) |
| **Whooley at initial antenatal visit**  **Q1 During the past month, have you often been bothered by feeling down, depressed or hopeless?**  Yes  No  Missing  **Q2 During the past month, have you often been bothered by little interest or pleasure in doing thing?**  Yes  No  Missing  **Positive to either Whooley Q1 or Q2**  Yes  No  Missing | 105 (6.0)  1518 (86.4)  134 (7.5)  75 (4.3)  1547 (88.1)  135 (7.7)  124 (7.1)  1499 (85.3)  134 (7.6) | 57 (7.4)  656 (85.3)  56 (7.3)  38 (4.9)  675 (87.8)  56 (7.3)  64 (8.3)  649 (84.4)  56 (7.3) | 48 (4.9)  862 (87.3)  78 (7.9)  37 (4.74)  872 (88.3)  79 (8.0)  60 (6.1)  850 (86.0)  78 (7.9) |
| **Body mass index at initial antenatal visit, kg/m^2^**  Mean (SD)  Median (IQR)  <18.5  18-5-24.9  25-29.9  30-34.9  35+  Missing | 27.2 (6.4)  26 (22.5-30.7)  34 (1.9)  704 (40.1)  513 (29.2)  272 (15.5)  224 (12.8)  10 (0.6) | 28.5 (7.0)  27 (23.4-32.5)  14 (1.8)  253 (32.9)  217 (28.2)  148 (19.3)  134 (17.4)  3 (0.4) | 26.2 (5.7)  25 (22-29)  20 (2.0)  451 (45.7)  296 (30.0)  124 (12.6)  90 (9.1)  7 (0.70 |
| **Smoking status at initial antenatal visit**  Yes (smoker)  No (gave up in the last 6 weeks)  No (gave up over 6 weeks ago)  No (never smoked)  Not recorded  Missing | 97 (5.5)  49 (2.8)  236 (13.4)  1359 (77.4)  15 (0.9)  1 (0.1) | 46 (6.0)  22 (2.9)  93 (12.1)  602 (78.3)  5 (0.7)  1 (0.1) | 51 (5.2)  27 (2.7)  143 (14.5)  757 (76.5)  10 (1.0)  0 (0.0) |
| **Parity**  First baby  Second baby  Third or more baby  Missing | 1076 (61.3)  451 (25.7)  230 (13.1)  0 (0.0) | 501 (65.2)  176 (22.9)  92 (12.0)  0 (0.0) | 575 (58.2)  275 (27.8)  138 (14.0)  0 (0.0) |
| **Number of live babies**  Singleton  Set of twins  Missing | 1712 (97.4)  45 (2.6)  0 (0.0) | 739 (96.1)  30 (3.9)  0 (0.0) | 973 (98.5)  15 (1.5)  0 (0.0) |

## Supplementary table 2: Labour, birth and postnatal at postnatal enrolment

| **Number of women** | **Totals** | **Hypertensive in pregnancy** | **Normotensive in pregnancy** |
| --- | --- | --- | --- |
| **Maternal outcomes: number of women** | **1757** | **769 (43.8)** | **988 (56.2)** |
| **Labour analgesia** *(non-exclusive)*  None  Oral drugs  Gas & Air  Intramuscular drugs  Alternative  Regional anaesthetic  General anaesthetic  Missing | 111 (6.3)  276 (15.7)  692 (60.6)  191 (10.9)  121 (6.9)  1228 (69.9)  45 (2.6)  1 (0.1) | 39 (5.1)  147 (19.1)  295 (38.4)  79 (10.3)  34 (4.4)  572 (74.4)  28 (3.6)  1 (0.1) | 72 (7.3)  129 (13.1)  397 (40.2)  112 (11.3)  87 (8.8)  656 (66.4)  17 (1.7)  0 (0.0) |
| **Perineal tear**  Graze/Laceration  1st degree tear  2nd degree tear  Episiotomy  3rd degree tear/ 4th degree tear | 72 (4.1)  79 (4.5)  350 (19.9)  282 (16.1)  36 (2.1) | 31 (4.0)  38 (4.9)  126 (16.4)  114 (14.8)  10 (1.3) | 41 (4.2)  41 (4.2)  224 (22.7)  168 (17.0)  26 (2.6) |
|  |  |  |  |
| **Perinatal outcomes: number of infants** | **1803** | **799 (44.3)** | **1004 (55.7)** |
| **Baby order**  Baby 1  Baby 2  Baby 3 | 1757 (97.5)  45 (2.5)  1 (0.1) | 769 (96.3)  30 (3.8)  0 (0.0) | 988 (98.4)  15 (1.5)  1 (0.1) |
| **Birth outcome**  Livebirth  Stillbirth | 1802 (99.9)  1(0.1) | 799 (100.0)  0 (0.0) | 1003 (99.9)  1(0.1) |

*Adjusted for clustering by twins

## Supplementary table 3: Study specific postnatal enrolment baseline characteristics

| **Number of women** | **Totals**  **1757** | **Women with hypertension**  **769 (43.8)** | **Women without hypertension**  **988 (56.2)** |
| --- | --- | --- | --- |
| **Number of women** | **1757** | **769 (43.8)** | **988 (56.2)** |
| **EQ-5D VAS score (0-100)**  Mean (SD)  Median (IQR) | 71.8 (18.8)  75 (60-85) | 69.2 (19.9)  70 (60-80) | 73.9 (17.6)  76 (65-88) |
| **EQ-5D index score (0.000-1)**  Mean (SD)  Median (IQR)  Mobility  No problems  Slight  Moderate  Severe  Unable/Extreme  Self-care  No problems  Slight  Moderate  Severe  Unable/Extreme  Usual activities  No problems  Slight  Moderate  Severe  Unable/Extreme  Pain / Discomfort  No pain/discomfort  Slight  Moderate  Severe  Extreme pain  Anxiety/Depression  Not anxious/depressed  Slight  Moderate  Severe  Extremely anxious/depressed | 0.778 (0.188)  0.809 (0.697-0.922)  826 (47.0)  576 (32.8)  273 (15.5)  54 (3.1)  28 (1.6)  1008 (57.4)  493 (28.1)  193 (11.0)  37 (2.1)  26 (1.5)  599 (34.1)  564 (32.1)  348 (19.8)  97 (5.5)  149 (8.5)  218 (12.4)  851 (48.4)  567 (32.3)  110 (6.3)  11 (0.6)  1339 (76.2)  332 (18.9)  70 (4.0)  13 (0.7)  3 (0.2) | 0.774 (0.191)  0.809 (0.697-0.922)  365 (47.5)  247 (32.1)  125 (16.3)  27 (3.5)  5 (0.7)  443 (57.6)  208 (27.1)  93 (12.1)  16 (2.1)  9 (1.2)  272 (35.4)  240 (31.2)  151 (19.6)  46 (6.0)  60 (7.8)  105 (13.7)  340 (44.2)  259 (33.7)  63 (8.2)  2 (0.3)  553 (71.9)  168 (21.9)  41 (5.3)  4 (0.5)  3 (0.4) | 0.780 (0.185)  0.812 (0.703-0.916)  461 (46.7)  329 (33.3)  148 (15.0)  27 (2.7)  23 (2.3)  565 (57.2)  285 (28.9)  100 (10.1)  21 (2.1)  17 (1.7)  327 (33.1)  324 (32.8)  197 (19.9)  51 (5.2)  89 (9.0)  113 (11.4)  551 (51.7)  308 (31.2)  47 (4.8)  9 (0.9)  786 (79.6)  164 (16.6)  29 (2.9)  9 (0.9)  0 (0.0) |
| **EPDS total score**  Mean (SD)  Median (IQR) | 6.7 (4.9)  6 (3-10) | 7.4 (5.1)  7 (3-11) | 6.1 (4.7)  5 (2-9) |
| **EPDS score ≥13** | 222 (12.6) | 126 (16.4) | 96 (9.7) |
| **Whooley at postnatal enrolment**  **Q1 During the past month, have you often been bothered by feeling down, depressed or hopeless?**  Yes  No  **Q2 During the past month, have you often been bothered by little interest or pleasure in doing thing?**  Yes  No  **Positive to either Whooley Q1 or Q2**  Yes  No | 302 (17.2)  1455 (82.8)  282 (16.1)  1475 (84.0)  396 (22.5)  1361 (77.5) | 163 (21.2)  606 (78.8)  147 (19.1)  622 (80.9)  211 (27.4)  558 (72.6) | 139 (14.1)  849 (85.9)  135 (13.7)  853 (86.3)  185 (18.7)  803 (81.3) |

## Supplementary table 4: Primary and main secondary outcomes at 3 months postnatal

| **Number of women** | **Totals** | **Hypertensive in pregnancy** | **Normotensive in pregnancy** |
| --- | --- | --- | --- |
| **Number of women** | **653** | **290 (44.4)** | **363 (55.6)** |
| **EQ-5D VAS score (0-100)**  Mean (SD)  Median (IQR) | 82.8 (13.3)  85 (79-90) | 82.2 (13.5)  85 (75-90) | 83.2 (13.1)  85 (80-90) |
| **EQ-5D index score (0.000-1)**  Mean (SD)  Median (IQR)  Mobility  No problems  Slight  Moderate  Severe  Unable/Extreme  Self-care  No problems  Slight  Moderate  Severe  Unable/Extreme  Usual activities  No problems  Slight  Moderate  Severe  Unable/Extreme  Pain / Discomfort  No problems  Slight  Moderate  Severe  Unable/Extreme  Anxiety/Depression  No problems  Slight  Moderate  Severe  Unable/Extreme | 0.935 (0.088)  0.942 (0.896-1)  598 (91.6)  51 (7.8)  3 (0.5)  1 (0.2)  0 (0.0)  638 (97.7)  13 (2.0)  2 (0.3)  0 (0.0)  0 (0.0)  554 (84.8)  82 (12.6)  13 (2.0)  3 (0.5)  1 (0.2)  451 (69.1)  180 (27.6)  20 (3.1)  2 (0.3)  0 (0.0)  451 (69.1)  147 (22.5)  46 (7.0)  7 (1.1)  2 (0.31) | 0.926 (0.093)  0.937 (0.887-1)  257 (88.6)  31 (10.7)  2 (0.7)  0 (0.0)  0 (0.0)  284 (97.9)  4 (1.4)  2 (0.7)  0 (0.0)  0 (0.0)  240 (82.8)  44 (15.2)  3 (1.0)  3 (1.0)  0 (0.0)  185 (63.8)  95 (32.8)  9 (3.1)  1 (0.3)  0 (0.0)  194 (66.9)  68 (23.5)  23 (7.9)  3 (1.0)  2 (0.7) | 0.943 (0.084)  1 (0.922-1)  341 (93.9)  20 (5.5)  1 (0.3)  1 (0.3)  0 (0.0)  354 (97.5)  9 (2.5)  0 (0.0)  0 (0.0)  0 (0.0)  314 (86.5)  38 (10.5)  10 (2.8)  0 (0.0)  1 (0.3)  266 (73.3)  85 (23.4)  11 (3.0)  1 (2.3)  0 (0.0)  257 (70.8)  79 (21.8)  23 (6.3)  4 (1.1)  0 (0.0) |
| **EPDS total score**  Mean (SD)  Median (IQR) | 6.2 (4.6)  5 (3-9) | 6.3 (4.8)  5.5 (3-9) | 6.1 (4.5)  5 (3-9) |
| **EPDS score ≥13** | 64 (9.8) | 34 (11.7) | 30 (8.3) |
| **Whooley at 3 months postnatal**  **Q1 During the past month, have you often been bothered by feeling down, depressed or hopeless?**  Yes  No  **Q2 During the past month, have you often been bothered by little interest or pleasure in doing thing?**  Yes  No  **Positive to either Whooley Q1 or Q2**  Yes  No | 161 (24.7)  492 (75.3)  109 (16.7)  544 (83.2)  183 (28.0)  470 (72.0) | 73 (25.2)  217 (74.8)  48 (16.6)  242 (83.5)  81 (27.9)  209 (72.1) | 88 (24.2)  275 (75.8)  61 (16.8)  302 (83.2)  102 (28.1)  261 (71.9) |
| **Morbidity**  Women with any morbidity  Women with any morbidity who consulted a health care professional  Extreme tiredness/exhaustion  Severe headaches/migraines  Back pain  Perineal wound infection  Caesarean wound infection  Breastfeeding problems  Leakage of urine  Leakage of stool  Other problems  **Frequency of these morbidities this week**  Extreme tiredness/exhaustion  Never  Rarely  Occasionally  Often  Severe headaches/migraines  Never  Rarely  Occasionally  Often  Back pain  Never  Rarely  Occasionally  Often  Perineal wound infection  Never  Rarely  Occasionally  Often  Caesarean wound infection  Never  Rarely  Occasionally  Often  Breastfeeding problems  Never  Rarely  Occasionally  Often  Leakage of urine  Never  Rarely  Occasionally  Often  Leakage of stool  Never  Rarely  Occasionally  Often  Other problems  Never  Rarely  Occasionally  Often | 582 (89.1)  378 (57.9)  252(38.6)  100 (15.3)  357 (54.7)  36 (5.5)  61 (9.3)  298 (45.6)  189 (29.0)  26 (4.0)  161 (24.7)  **N=252**  38 (15.1)  55 (21.8)  113 (44.8)  46 (18.3)  **N=100**  32 (32.0)  18 (18.0)  71 (37.0)  13 (13.0)  **N=357**  26 (7.3)  72 (20.2)  174 (48.7)  85 (23.8)  **N=36**  27 (75.0)  2 (5.6)  6 (16.7)  1 (2.8)  **N=59**  38 (64.4)  6 (10.2)  10 (17.0)  5 (8.5)  **N=259**  133 (51.4)  55 (21.3)  45 (17.4)  26 (10.0)  **N=189**  51 (27.0)  68 (36.0)  48 (25.4)  22 (11.6)  **N=26**  17 (65.4)  5 (19.2)  2 (7.7)  2 (7.8)  **N=161**  60 (37.3)  23 (14.3)  36 (22.4)  42 (26.1) | 260 (89.7)  171 (59.0)  120 (41.4)  55 (19.0)  168 (57.9)  18 (6.2)  33 (11.4)  120 (41.4)  83 (28.6)  10 (3.5)  73 (25.2)  **N=120**  13 (10.8)  24 (20.0)  57 (47.5)  26 (21.7)  **N=55**  15 (27.3)  10 (18.2)  22 (40.0)  8 (14.6)  **N=168**  12 (7.1)  26 (15.5)  84 (50.0)  46 (27.4)  **N=18**  13 (72.2)  2 (11.1)  2 (11.1)  1 (5.6)  **N=32**  21 (65.6)  2 (6.3)  6 (18.8)  3 (9.4)  **N=105**  54 (51.4)  23 (21.9)  15 (14.3)  13 (12.4)  **N=83**  21 (25.3)  29 (34.9)  21 (25.3)  12 (14.5)  **N=10**  5 (50.0)  3 (30.0)  0 (0.0)  2 (20.0)  **N=73**  26 (35.6)  12 (16.4)  17 (23.3)  18 (24.7) | 322 (88.7)  207 (57.0)  132 (36.4)  45 (12.4)  189 (52.1)  18 (5.0)  28 (7.7)  178 (49.0)  106 (29.2)  16 (4.4)  88 (24.2)  **N=132**  25 (18.9)  31 (23.5)  56 (42.4)  20 (15.2)  **N=45**  17 (37.8)  9 (17.8)  15 (33.3)  5 (11.1)  **N=189**  14 (7.4)  46 (24.3)  90 (47.6)  39 (20.6)  **N=18**  14 (77.8)  0 (0.0)  4 (22.2)  0 (0.0)  **N=27**  17 (63.0)  4 (14.8)  4 (14.8)  2 (7.4)  **N=154**  79 (51.3)  32 (20.8)  30 (19.5)  13 (8.4)  **N=106**  30 (28.3)  39 (36.8)  27 (25.5)  10 (9.4)  **N=16**  12 (75.0)  2 (12.5)  2 (12.5)  0 (0.0)  **N=88**  34 (28.6)  11 (12.5)  19 (21.6)  24 (27.3) |
| **Ever breastfeed baby**  Yes  No  Missing | 586 (89.7)  67 (10.3)  0 (0.0) | 261 (90.0)  29 (10.0)  0 (0.0) | 325 (89.5)  38 (10.5)  0 (0.0) |
| **First feed (of those who ever breastfed)**  Breastmilk (from breast)  Breastmilk (expressed)  Infant formula only  Other | 438 (74.7)  102 (17.4)  33 (5.6)  13 (2.2) | 179 (68.6)  52 (19.9)  20 (7.7)  10 (3.8) | 259 (79.7)  50 (15.4)  13 (4.0)  3 (0.9) |
| **Current feeding (of those who ever breastfed)**  Breastmilk (from breast or expressed)  Infant formula only  Breastmilk and infant formula  Other | 305 (52.1)  155 (26.5)  126 (21.5)  0 (0.0) | 129 (49.4)  69 (26.4)  63 (24.1)  0 (0.0) | 176 (54.2)  86 (26.5)  63 (19.4)  0 (0.0) |

## Supplementary table 5: Postnatal care at 3 months postnatal and opinions on future pregnancies (self-reported)

|  | **Totals** | **Hypertensive in pregnancy** | **Normotensive in pregnancy** |
| --- | --- | --- | --- |
| **Number of women** | **653** | **290 (44.4)** | **363 (55.6)** |
| **BP measured in hospital**  Yes  No  Missing | 619 (94.8)  21 (3.2)  13 (2.0) | 274 (94.5)  8 (2.8)  8 (2.8) | 345 (95.0)  13 (3.6)  5 (1.4) |
| **BP medication taken between birth and 6 weeks postnatal**  Yes  No  Missing  BP meds reviewed before left hospital  BP meds review since left hospital | 208 (31.9)  432 (66.2)  13 (2.0)  **N=208**  165 (79.3)  163 (78.4) | 194 (66.9)  88 (30.3)  8 (2.8)  **N=194**  155 (79.9)  154 (79.4) | 14 (3.9)  344 (94.8)  5 (1.4)  **N=14**  10 (71.4)  9 (64.3) |
| **Postnatal information provision on:**  Physical health  Mental health  Breastfeeding  Safety of BP medications when breastfeeding  Sign and symptoms of high BP  Contact information in case of problems with your health, or your baby’s health, when at home  Long term consequences of high BP in pregnancy  Self-management or lifestyle changes to help manage BP | 536 (82.1)  532 (81.5)  587 (89.9)  277 (42.4)  620 (95.0) | 232 (80.0)  233 (80.3)  255 (87.9)  100 (49.3)  173 (59.7)  276 (95.2)  120 (47.2)  92 (36.7) | 304 (83.8)  299 (82.4)  332 (91.5)  104 (28.7)  344 (94.8)  N/A  N/A |
| **Postnatal visits at home**  One or more postnatal visits  Mean number of visits (SD)  Median number of visits (IQR)  BP measured at every visit  BP measured at some visits  BP never measured | 505 (77.3)  3.4 (2.2)  2 (2-3)  **N=505**  172 (34.1)  137 (27.1)  196 (38.8) | 236 (81.4)  3.7 (2.5)  3 (2-2)  **N=236**  115 (48.7)  65 (27.5)  56 (23.7) | 269 (74.1)  3.2 (1.9)  3 (2-4)  **N=269**  57 (21.2)  72 (26.8)  140 (52.0) |
| **Postnatal visits at hospital/clinic**  One or more postnatal visits  Mean number of visits (SD)  Median number of visits (IQR)  BP measured at every visit  BP measured at some visits  BP never measured | 215 (32.9)  2.3 (2.8)  2 (1-3)  **N=215**  110 (51.2)  28 (13.0)  77 (35.8) | 104 (35.9)  2.6 (3.6)  2 (1-3)  **N=104**  67 (64.4)  13 (12.5)  24 (23.1) | 111 (30.6)  2.1 (1.9)  1 (1-2)  **N=111**  43 (38.7)  15 (13.5)  53 (47.8) |
| **Postnatal visits at the GP**  One or more postnatal visits  Mean number of visits (SD)  Median number of visits (IQR)  BP measured at every visit  BP measured at some visits  BP never measured | 466 (71.4)  2.1 (3.1)  1 (1-2)  **N=466**  291 (62.5)  85 (18.2)  90 (19.3) | 222 (76.6)  2.5 (3.3)  2 (1-3)  **N=222**  163 (73.4)  36 (16.2)  23 (10.4) | 244 (67.2)  1.8 (2.7)  1 (1-2)  **N=244**  128 (52.5)  49 (20.1)  67 (27.5) |
| **Postnatal visits at other NHS facilities**  One or more postnatal visits  Mean number of visits (SD)  Median number of visits (IQR)  BP measured at every visit  BP measured at some visits  BP never measured | 31 (4.8)  5.6 (15.8)  2 (2-1)  **N=31**  3 (9.7)  1 (3.2)  27 (87.1) | 12 (4.1)  3.1 (3.1)  2 (2-1)  **N=12**  2 (16.7)  1 (8.3)  9 (75.0) | 19 (5.2)  7.2 (20.1)  2 (4-1)  **N=19**  1 (5.3)  0 (0.0)  18 (94.7) |
| **Readmitted to hospital**  Yes  No  Missing | 58 (8.9)  582 (89.1)  13 (2.0) | 38 (13.1)  244 (84.1)  8(2.8) | 20 (5.5)  338 (93.1)  5 (1.4) |
| **Readmitted to hospital due to BP**  Yes  No  Not applicable/Missing | 28 (4.3)  30 (4.6)  595 (91.1) | 24 (8.3)  14 (4.8)  252 (86.9) | 4 (1.1)  16 (4.4)  343 (94.5) |
| **GP postnatal check attendance**  No  Yes accepted  Yes declined  Missing  BP measured | 39 (6.0)  584 (89.4)  17 (2.6)  13 (2.0)  **N=584**  459 (78.6) | 19 (6.6)  258 (89.0)  5 (1.7)  8 (2.8)  **N=258**  214 (83.0) | 20 (5.5)  326 (89.8)  12 (3.3)  5 (1.4)  **N=326**  245 (75.2) |
| **Any self-monitoring of BP since birth**  Yes  No  Missing | 154 (23.6)  486 (74.4)  13 (2.0) | 125 (43.1)  157 (54.1)  8 (2.8) | 29 (8.0)  329 (90.6)  5 (1.4) |
| **Regular self-monitoring of BP since birth**  Yes  No  Not applicable/Missing | 79 (12.1)  75 (11.5)  449 (76.4) | 72 (24.8)  53 (18.3)  165 (56.9) | 7 (1.9)  22 (6.1)  334 (92.0) |
| **Opinion of postnatal care (overall)**  Excellent  Very good  Good  Fair  Poor  NA  Missing | 177 (27.1)  228 (34.9)  143 (21.9)  66 (10.1)  25 (3.8)  1 (0.2)  13 (2.0) | 92 (31.7)  87 (30.0)  53 (18.3)  36 (12.4)  13 (4.5)  1 (0.3)  8 (2.8) | 85 (23.4)  141 (38.8)  90 (24.8)  30 (8.3)  12 (3.3)  0 (0.0)  5 (1.4) |
| **Opinion of postnatal care in hospital**  Excellent  Very good  Good  Fair  Poor  NA  Missing | 245 (37.5)  205 (31.4)  90 (13.8)  59 (9.0)  38 (5.8)  3 (0.5)  13 (2.0) | 112 (38.6)  85 (29.3)  39 (13.5)  27 (9.3)  17 (5.9)  2 (0.7)  8 (2.8) | 122 (36.6)  120 (33.1)  51 (14.1)  32 (8.8)  21 (5.8)  1 (0.3)  5 (1.4) |
| **Opinion of postnatal care at home**  Excellent  Very good  Good  Fair  Poor  NA  Missing | 185 (28.3)  180 (27.6)  162 (24.8)  66 (10.1)  28 (4.3)  19 (2.9)  13 (2.0) | 95 (32.8)  66 (22.8)  66 (22.8)  33 (11.4)  14 (4.8)  8 (2.8)  8 (2.8) | 90 (24.8)  114 (31.4)  96 (26.5)  33 (9.1)  14 (3.9)  11 (3.0)  5 (1.4) |
| **Are you likely to consider having a future pregnancy?**  Yes  No  Missing | 421 (64.5)  218 (33.4)  14 (2.1) | 192 (66.2)  90 (31.0)  8 (2.8) | 229 (63.1)  128 (35.3)  6 (1.7) |
| **Are you currently pregnant?**  Yes  No  Missing | 3 (0.5)  636 (97.4)  14 (2.1) | 2 (0.7)  280 (96.6)  8 (2.8) | 1 (0.3)  356 (98.1)  6 (1.7) |
| **Has your recent pregnancy/birth/postnatal changed your family plans?**  Yes  No  Missing | 206 (31.6)  433 (66.3)  14 (2.1) | 107 (36.9)  175 (60.3)  8 (2.8) | 99 (27.3)  258 (71.1)  6 (1.7) |

## Supplementary table 6: Maternal demographics at postnatal enrolment by responder status

|  | **Totals** | **Responder^$^** | **Non-responder** |
| --- | --- | --- | --- |
| **Number of women** | **1757** | **654 (37.2)** | **1,103 (62.8)** |
| **Number of women with hypertension** | 769 (43.8) | 290 (44.3) | 479 (43.4) |
| **Mean (SD) maternal age, years** | 32.7 (5.5) | 33.3 (5.2) | 32.4 (5.6) |
| **Self-reported ethnicity groupings**  Black/black mixed  Asian/Asian mixed  White  Other  Missing | 263 (15.0)  187 (10.7)  1230 (70.0)  75 (4.3)  2 (0.1) | 68 (10.4)  56 (8.6)  505 (77.2)  23 (3.5)  2 (0.3) | 195 (17.7)  131 (11.9)  725 (65.7)  52 (4.7)  0 (0.0) |
| **Education level beyond secondary (self-reported)** | 1375 (78.3) | 559 (85.5) | 816 (74.0) |
| **Currently employed (self-reported)** | 1321 (75.2) | 523 (80.0) | 798 (72.4) |
| **Living with others (self-reported)** | 1554 (88.5) | 583 (89.1) | 971 (88.0) |
| **Any risk factors for hypertension (self-reported)** | 1395 (79.4) | 533 (81.5) | 862 (78.2) |
| **Any mental health condition (self-reported)** | 307 (17.5) | 124 (19.0) | 183 (16.6) |
| **Positive to either Whooley questions at initial antenatal visit**  Missing | 124 (7.1)  134 (7.6) | 39 (6.0)  54 (8.3) | 85 (7.7)  80 (7.3) |
| **Median (IQR) body mass index at initial antenatal visit, kg/m^2^** | 26 (22.5-30.7) | 25.1 (22.2-30.6) | 26 (22.6-31) |
| **Current smoker at initial antenatal visit** | 97 (5.5) | 22 (3.4) | 75 (6.8) |
| **First baby** | 1076 (61.3) | 426 (65.1) | 650 (58.9) |
| **Singleton delivery** | 1712 (97.4) | 633 (96.8) | 1079 (97.8) |
| **Intended mode of birth**  Spontaneous vaginal birth  Elective c-section  Planned induction (vaginal birth)  Not discussed - delivered early  Not discussed – other  Missing | 1062 (60.4)  304 (17.3)  289 (16.5)  24 (1.4)  78 (4.4)  0 (0.0) | 399 (61.0)  115 (17.6)  108 (16.5)  4 (0.6)  28 (4.3)  0 (0.0) | 663 (60.1)  189 (17.1)  182 (16.4)  20 (1.8)  50 (4.5)  0 (0.0) |

**^$^**Responder = a woman who completed the 3-month questionnaire

## Supplementary table 7: Characteristics of labour, birth and post-delivery postnatal stay at postnatal enrolment by responder status

| **Number of women** | **Totals** | **Responder^$^** | **Non-responder** |
| --- | --- | --- | --- |
| **Maternal outcomes: number of women** | **1757** | **654 (37.2)** | **1,103 (62.8)** |
| **Number of women with hypertension** | 769 (43.8) | 290 (44.3) | 479 (43.4) |
| **Use of regional anaesthetic in labour** | 1228 (69.9) | 455 (69.6) | 773 (70.1) |
| **Second/third/fourth degree perineal tear or episiotomy** | 668 (38.0) | 267 (40.8) | 401 (36.4) |
| **Median (IQR) length of postnatal inpatient stay, days** | 3 (2-4) | 3 (2-4) | 3 (2-4) |
| **Admission to either high dependency unit or intensive care unit**** | 181 (10.3) | 85 (13.0) | 96 (8.7) |
| **Median (IQR) length of stay in high dependency unit or intensive care unit, days** | 1 (1-1) | 1 (1-1) | 1 (1-1) |
|  |  |  |  |
| **Perinatal outcomes: number of infants** | **1803** | **675 (37.4)** | **1128 (62.6)** |
| **Number of babies to women with hypertension** | 799 (44.3) | 303 (44.9) | 496 (44.0) |
| **Livebirths** | 1802 (99.9) | 675 (100.0) | 1127 (99.9) |
| **Mode of delivery**  Vaginal  Forceps  Ventouse  Pre-labour c-section  In-labour c-section  Missing | 657 (36.4)  168 (9.3)  137 (7.6)  462 (25.6)  379 (21.0)  0 (0.0) | 235 (34.8)  71 (10.5)  56 (8.3)  181 (26.8)  132 (19.6)  0 (0.0) | 422 (37.4)  97 (8.6)  81 (7.2)  281 (24.9)  247 (21.9)  0 (0.0) |
| **Mean (SD) gestational age at birth, weeks** | 38.8 (2.3) | 38.8 (2.3) | 38.8 (2.3) |
| **Mean (SD) birthweight (kg)** | 3.198 (0.671) | 3.197 (0.681) | 3.199 (0.666) |
| **Small for Gestational Age** (<10^th^ birthweight centile) | 184 (10.2) | 64 (9.5) | 120 (10.6) |
| **Mean (SD) length of infant inpatient stay, days** | 4.2 (6.7) | 4.4 (6.8) | 4.1 (6.7) |
| **Baby (ies) admitted to the neonatal unit** | 230 (12.8) | 91 (13.5) | 139 (12.3) |
| **Mean (SD) length of stay in neonatal unit, days** | 9.7 (9.6) | 9.8 (9.9) | 9.6 (9.4) |

*Adjusted for clustering by twins **Includes post section recovery **^$^**Responder = a woman who completed the 3-month questionnaire

## Supplementary table 8: Study specific outcomes at postnatal enrolment by responder status

|  | **Totals** | **Responder^$^** | **Non-responder** |
| --- | --- | --- | --- |
| **Number of women** | **1757** | **654 (37.2)** | **1,103 (62.8)** |
| **EQ-5D VAS score (0-100)**  Mean (SD)  Median (IQR) | 71.8 (18.8)  75 (60-85) | 72.0 (17.1)  75 (64-80) | 71.7 (19.7)  75 (60-85) |
| **EPDS score ≥13**  N (%) | 222 (12.6) | 74 (11.3) | 148 (13.4) |
| **Positive to either Whooley Q1 or Q2**  **at postnatal enrolment** | 396 (22.5) | 266 (24.1) | 130 (19.9) |

**^$^**Responder = a woman who completed the 3-month questionnaire
